# Potential PGPR Properties of Cellulolytic, Nitrogen-Fixing, Phosphate-Solubilizing Bacteria in Rehabilitated Tropical Forest Soil

**DOI:** 10.3390/microorganisms8030442

**Published:** 2020-03-20

**Authors:** Amelia Tang, Ahmed Osumanu Haruna, Nik Muhamad Ab. Majid, Mohamadu Boyie Jalloh

**Affiliations:** 1 Faculty of Agriculture and Food Sciences, Universiti Putra Malaysia Bintulu Campus, Bintulu 97008, Sarawak, Malaysia; ameliatang18@gmail.com; 2 Institute of Tropical Agriculture and Food Security (ITAFoS), Universiti Putra Malaysia, Serdang 43400, Selangor, Malaysia; 3 Institute of Tropical Forestry and Forest Products (INTROP), Universiti Putra Malaysia, Serdang 43400, Selangor, Malaysia; nik@upm.edu.my; 4 Faculty of Sustainable Agriculture, Universiti Malaysia Sabah, Sandakan Branch, Locked Bag No. 3, Sandakan 90509, Sabah, Malaysia; mbjalloh@ums.edu.my

**Keywords:** rehabilitated forest, functional bacteria, cellulolytic activities, nitrogen-fixation activities, phosphate-solubilization activities, IAA production

## Abstract

In the midst of the major soil degradation and erosion faced by tropical ecosystems, rehabilitated forests are being established to avoid the further deterioration of forest lands. In this context, cellulolytic, nitrogen-fixing (N-fixing), phosphate-solubilizing bacteria are very important functional groups in regulating the elemental cycle and plant nutrition, hence replenishing the nutrient content in forest soils. As is the case for other potential plant growth-promoting (PGP) rhizobacteria, these functional bacteria could have cross-functional abilities or beneficial traits that are essential for plants and can improve their growth. This study was conducted to isolate, identify, and characterize selected PGP properties of these three functional groups of bacteria from tropical rehabilitated forest soils at Universiti Putra Malaysia Bintulu Sarawak Campus, Malaysia. The bacteria were isolated based on their colonial growth on respective functional media, identified using both molecular and selected biochemical properties, and were assessed for their functional quantitative activities as well as PGP properties based on seed germination tests and indole-3-acetic acid (IAA) production. Out of the 15 identified bacterial isolates that exhibited beneficial phenotypic traits, a third belong to the genus *Burkholderia* and a fifth to *Stenotrophomonas* sp., with both genera consisting of members from two different functional groups. The results of the experiments confirm the multiple PGP traits of some selected bacterial isolates based on their respective high functional activities, root and shoot lengths, and seedling vigor improvements when bacterized on mung bean seeds, as well as significant IAA production. The results of this study suggest that these functional bacterial strains could potentially be included in bio-fertilizer formulations for crop growth on acid soils.

## 1. Introduction

Although being vast in carbon and biodiversity, tropical ecosystems are facing major deforestation issues, leading to widespread soil erosion, which has garnered much recent attention [1,2,3]. As soil erosion episodes escalate, the resultant soil loss overrides soil establishment, which results in diminishing soil resources and the ecosystem supports they render [4,5]. This is manifested particularly as low productivity and farming sustainability on these degraded soils [6]. Planted forests or rehabilitation efforts are thus being undertaken to avoid further deterioration of forest lands. 

Bacteria biodiversity and composition in soils is vast, resulting in major roles in principal soil processes that regulate whole terrestrial ecosystem operations. This occurs in nutrient and geochemical cycles, including for C, N, S, and P [7,8,9]. In plant litter mass, 20%–30% constitutes cellulose, making it the most abundant biopolymer [10]. The decomposition of cellulose is primarily due to soil microorganisms’ activities, which assimilate the simple sugars resulting from the reduction process of complex polysaccharides [11,12]. Despite the generally better capabilities of fungi over bacteria in cellulose decomposition in soils [13,14], these capabilities are also expressed by some phylogenetically different taxa of bacteria [11]. Previous reports have emphasized bacterial taxa, which have been recently discovered to possess cellulolytic capabilities, where they were formerly not known to have the capability to reduce cellulose [15,16]. Therefore, research on cellulolytic bacterial diversity in tropical soils is necessary. 

The nitrogen needs of plants are supported by means of organic compound degradation and the atmospheric deposition of N and BNF, wherein BNF supplies 97% of the natural N reserves [17] via *Bacteria* and *Archaea* [18,19]. The N allocation in tropical ecosystems contributed to by N-fixing microorganisms (diazotrophs), existing mostly in free-living conditions, can range from 12.2 to 36.1 kg ha^−1^ year^−1^ [20]. Considering the importance of free-living diazotrophic communities to ecosystem processes, there is a need to broaden our knowledge on their diversity in rehabilitated tropical forests due to a dearth of such information.

In most tropical soils, which are usually very acidic, P is one of the most common deficient macronutrients, due to high soil Al and/or Fe levels, in which P is fixed as insoluble mineral complexes, causing the unavailability of P for root uptake [21]. Apart from chemical amendments, microorganisms have also been applied over the years to improve nutrient availability, especially P in soils, as well as for alleviating Al toxicity [22]. Furthermore, tropical acid soils support only limited acid-tolerant plant species and microbes. In soils, phosphate-solubilizers are predominatly bacteria compared to fungi with values of 1%–50% and 0.1%–0.5%, of the total population, respectively [23]. Bacterial strains comprising *Pseudomonas*, *Bacillus*, and *Rhizobium* have been cited to be the dominant phosphate-solubilizers [24]. In addition, *Burkholderia* strains and species that occur in broadly different natural niches have been frequently reported as plant-associated bacteria, despite their prevalence as free-living in the rhizosphere, as well as epiphytic or endophytic, obligate endosymbionts, or phytopathogens [25]. The PSB can release substantial amounts of organic acids crucial for fixing Al via a chelation process, thus reducing Al toxicity to plant roots in highly weathered soils [26].

As with other potential PGPB, these functional bacteria could have cross-functional abilities or beneficial traits, apart from their primary functional abilities, such as the production of plant growth-promoting (PGP) phytohormones, polysaccharides, and organic acids that are essential for plant growth and development [22]. In addition, the indole-3-acetic acid (IAA) produced by bacteria stimulates shoot elongation and/or in particular root structures [27], which could result in more efficient elemental nutrient acquisition by host plants, leading to higher plant growth and development. The adoption of functional bacterial strains with multiple functional and PGP traits in crop production could promote sustainable crop productivity, offer an option for minimizing costs related to chemical fertilizer application and reduce pollution risks from excessive application of chemical fertilizers.

However, the isolation and characterization of these three particular types of potential functional bacteria from tropical soils of rehabilitated forests remain scarce. Thus, the aims of this study were to: 1) isolate and identify cellulolytic, N-fixing, and phosphate-solubilizing bacteria from a rehabilitated tropical forest soil, and 2) characterize cellulolytic, N-fixing, and phosphate-solubilizing bacteria for their respective functional activities, such as IAA production, early plant growth promotion and some phenotypic and biochemical properties. 

## 2. Materials and Methods

### 2.1. Soil Sampling and Physico-Chemical Properties

Soil samples were collected from a rehabilitated forest site at Universiti Putra Malaysia Bintulu Sarawak Campus, Malaysia, through random sampling method using aseptic techniques near the rhizospheric zones of planted trees. Soil samples were collected at 0–10 cm depth using an auger for various analyses. The soil samples were mixed thoroughly and kept in tightly sealed plastic bags and stored at 4 °C (for less than three days before bacterial analysis).

The soil at the study area belonged to Bekenu and Nyalau series (Ultisols) and was characterized as well-drained, red–yellow to yellow, siliceous, isohyperthermic fine loamy *Typic Tualemkuts* based on the USDA-Soil Taxonomy Classification System. The soil is a sandy loam texture containing 69.18% sand, 13.27% silt, and 17.55% clay. The soil is acidic with pH 4.71 and a CEC value of 8.13 cmol_c_ kg^−1^. The exchangeable K, Ca, and Mg contents were 0.10, 6.68, and 45.33 cmol_c_ kg^−1^, respectively. The total N, total C, and organic matter content were 0.13, 2.60, and 4.48%, respectively. The available P, exchangeable ammonium, and available nitrate in the soil were 6.22, 42.73, and 1.40 mg kg^−1^, respectively. 

### 2.2. Isolation and Plate Assays of Functional Bacteria

Five grams of fresh soil was mixed into 100 mL of sterile distilled water and shaken at 150 rpm for 24 h. The supernatant was serially diluted by ten-folds, and 0.1 mL aliquots were spread over Petri dishes containing the respective selective media in triplicates.

Nitrogen-fixing bacterial colonies were isolated on N-free malate (Nfb) medium [28] with some modifications. Details of the medium per liter were as follows: 0.4 g of KH_2_PO_4_, 0.1 g of K_2_HPO_4_, 0.2 g of MgSO_4_.7H_2_O, 0.1 g of NaCl, 0.02 g of CaCl_2_, 0.01 g of FeCl_3_, 0.002 g of MoO_4_Na.2H_2_O, 5.0 g of sodium malate, and 5 mL of bromothymol blue with 0.5% alcohol solution and 20.0 g of agar, with whole content adjusted to pH 7. The colonies that fix N_2_ alkalinize the culture medium (blue) because of ammonia production.

Phosphate-solubilizing bacterial colonies were isolated on tricalcium phosphate as insoluble phosphate-containing media to differentially isolate phosphate-solubilizing bacteria based on the formation of clear zones around their colonies [29,30,31,32] with some modifications. All media had 50 mL of a salts solution (MgCl_2_·6H_2_O, 100 g L^−1^; MgSO_4_·7H_2_O, 5 g L^−1^; KCl, 4 g L^−1^; (NH_4_)_2_SO_4_, 2 g L^−1^), 10 g of sucrose as C source, 20 g L^−1^ of agar and 2.5 g of tricalcium phosphate, Ca_3_(PO_4_)_2_, with 6 mL L^−1^ of bromophenol blue in 0.4% ethanol solution added to the media and content pH adjusted to 7.

Cellulolytic bacterial colonies were isolated on modified cellulose-Congo red agar from Hendricks et al. [33] per liter, comprising: 0.50 g of K_2_HPO_4_, 0.25 g of MgSO_4_, 1.88 g of cellulose microgranular powder, 0.20 g of Congo red, 5.00 g of agar, 2.00 g of gelatin, 100 mL of soil extract, and 900 mL of tap water (used to provide essential trace elements for soil bacteria), with content adjusted to pH 7. The colonies exhibiting zones of clearing would indicate cellulose-hydrolyzing activities.

After spread-plating, all media were incubated at 30 °C. Some colonies exhibiting large and instant blue halo or clearing zones on respective media were isolated, purified on new respective selective media, sub-cultured on agar slants, and stored at 4 °C for further studies. Plate assays of selected functional bacterial isolates were also done by spot-inoculating cultures in triplicates on the respective aforementioned media and incubated at 30 °C over 10 day-periods prior to the assessment of their functional activity efficiency, based on calculations by ratio of total diameter (colony and halo or clearing zone around colonies) and colony diameter [34].

### 2.3. Quantitative Assays for Functional Activities

#### 2.3.1. Quantitative Assay for Cellulase Activity

Cellulolytic isolates were cultured in nutrient broth, shaken at 120 rpm at 30 °C for 16 h, and the size of the pre-inoculum culture for cellulase assay was fixed to OD_600_ = 0.5 (10^6^ to 10^7^ cfu mL^−1^). A total of 1 mL of each pre-inoculum was seeded in 40 mL cellulose broth as a basal medium. The composition of the cellulose broth per liter was: 5.0 g L^−1^ cellulose microgranular, 2.5 g L^−1^ NaNO_3_, 1.0 g L^−1^ KH_2_PO_4_, 0.6 g L^−1^ MgSO_4_·7H_2_O, 0.1 g L^−1^ NaCl, 0.1 g L^−1^ CaCl_2_·6H_2_O, 0.01 g L^−1^ FeCl_3_, 2.0 g L^−1^ gelatin and 0.1 g L^−1^ yeast extract with pH 6.8–7.2 (modified from Lu et al. [35]). The inoculated broth was incubated at 30 °C under agitation at 180 rpm and CMCase activity was analyzed for up to 5 days. For CMCase activity, 0.5 mL culture filtrate from broth as crude enzyme source, or supernatant cellulase, also specified as CMCase, was incubated with 1 mL of 1% carboxymethylcellulose in 0.1 M citrate buffer, pH 5 at 40 °C in agitation for 30 min. The amount of reducing sugars (glucose) was measured using the Somogyi-Nelson method [36,37] at 520 nm. One unit of enzyme activity (IU mL^−1^) is equivalent to the amount of enzyme per mL of culture filtrate that liberates 1 µg of glucose per minute [38].

#### 2.3.2. Quantitative Assay for In Vitro BNF Activity

The procedure for quantitative estimation of BNF activity was modified from those described by Soares et al. [39]. Each N-fixing isolate was grown as a pre-inoculum in nutrient broth under agitation at 120 rpm at 30 °C for 16 h. Optical density was used to adjust the inoculum size [OD_600_ = 0.5 is equivalent to 10^6^ to 10^7^ colony forming units (cfu) mL^−1^]. The inoculum was then transferred (100 µL) to new media, NFb broth (containing similar composition as Nfb medium with the exception of agar). The inoculated media was incubated at 30 °C under constant agitation for 72 h for N_2_ fixation quantification. A control medium was also included by using a 100 µL nutrient broth as inoculant. The amount of N_2_ fixation was measured by sulfur digestion and distilled with NaOH 10 mol L^−1^, as described by Bremner and Keeney [40]. There were triplicates of the preparation. The samples were digested with 2 mL of concentrated H_2_SO_4_ (*d* = 1.84), 1 mL of H_2_O_2_ 30% and 0.7 g of the digestion mixture (100 g Na_2_SO_4_ + 10 g CuSO_4_.5H_2_O + 1 g selenium) at 350–375 °C. The products of the digestion were distillated with 10 mL of NaOH 10 mol L^−1^, and the ammonia trapped in 10 mL of 2% boric acid was measured by titration with 0.0025 mol L^−1^ H_2_SO_4_. 

#### 2.3.3. Quantitative Assay for Inorganic Phosphate-solubilization Activity

The quantitative estimation of inorganic phosphate-solubilization was done with some modifications from Delvasto et al. [41]. Pre-inoculum cultures were prepared earlier by inoculating phosphate-solubilizing isolates in nutrient broth under agitation at 120 rpm at 30 °C for 16 h. The inoculum size was adjusted to OD_600_ = 0.5 (10^6^ to 10^7^ cfu mL^−1^). Prior to the assay, pre-inoculum cultures were washed 3 times and resuspended using saline solution 0.85% NaCl and one mL isolate suspension was inoculated into a 100 mL liquid medium containing insoluble tricalcium phosphate, as in the solid medium but without agar. Inoculated broth was incubated at 30 °C under agitation at 180 rpm for up to 5 days. Saline solution without inoculant in the broth was left as the control. Soluble P content in culture filtrates was determined using the blue method [42] and UV-vis spectrophotometer at 882 nm. The final pH of the liquid medium was measured using a pH meter. All values of pH and P concentrations of the samples were measured in triplicates. 

#### 2.3.4. Morphological and Physiological Characterization of Selected Isolates

Selected isolates were individually studied for morphological and phenotypic properties. They were examined microscopically using a spore-staining procedure according to the Schaeffer-Fulton method and the standard Gram staining procedure described by Cappuccino and Sherman [43]. Biochemical tests and physiological activities, or differential growth tests, were also carried out. Citrate utilization was determined on Simmons citrate agar; hydrogen sulfide production, along with lactose and glucose fermentation were determined on Kligler Iron Agar; Gram negative strains and lactose fermentation were detected using Mac Conkey agar; gelatinase production, starch hydrolysis, casein proteolysis, lipase production, and indole production were each determined using nutrient gelatine, starch agar, skim milk agar, Tween 80 agar and BD’s Indole Reagent Droppers (modified Kovacs’ reagent) via the standard tube method, respectively. Catalase test was done using 3% hydrogen peroxide solution. A motility test was conducted on Biolife’s Motility Medium, whereas the nitrate reduction test was done using the test kit by Fluka (Fluka 73426, Sigma-Aldrich, Germany). The aforementioned physiological and biochemical test results were processed and compared with the results of systematic bacteriology identification according to Bergey’s Manual of Determinative Bacteriology and Bergey’s Manual of Systematic Bacteriology [44,45]. 

### 2.4. Molecular Method for Bacterial Identification

#### 2.4.1. Deoxyribonucleic Acid Extraction

Bacterial genomic DNA was extracted using the sodium dodecyl sulfate (SDS) method. Bacterial inoculum from overnight streaked culture plate was recultured overnight in 1.5 mL of nutrient broth in capped tubes with one control (broth without inoculum) in the water bath shaker at 30 °C. The 1.5 mL cell suspension was centrifuged at 13,040× *g* for 3 min at 4 °C. After removing the supernatant, the cells were resuspended or washed with 180 µL of TE buffer (10 mM Tris, 1mM EDTA, pH 8.0) by vortex-mixing and incubated on ice for 5 min. A total of 75 µL of 2% SDS solution was added, followed by an invert-mixing step and incubation on ice for 5–10 min. Then, 250 µL of 3 M potassium acetate was added, followed by gentle invert-mixing and incubation on ice for 5 min. The sample was centrifuged at 13,040× *g* for 5 min at 4 °C. The supernatant containing bacterial DNA was carefully transferred to a clean 1.5 mL tube on ice. Absolute ethanol was then added at approximately two times the volume of the supernatant in the same tube, invert-mixed followed by centrifugation at 12,000× *g* for 10 min at 4 °C. The supernatant was discarded, and 1 mL of 75% ethanol was added to the DNA pellet. The pellet was resuspended by gently pipette-mixing the suspension followed by centrifugation at 12,000× *g* for 10 min at 4 °C. The supernatant was discarded, and the pellet was air-dried. Finally, 20 µL of TE buffer (10 mM Tris, 1mM EDTA, pH 8.0) was added, pipette-mixed gently, and stored at −20 °C.

#### 2.4.2. Polymerase Chain Reaction Amplification of Extracted DNA

The amplification of DNA extraction products was performed in a final volume of 25 µL each. For each reaction, 1.25 µL 0.5 µM for both of the primers (16S-F and 16S-R1), 2.5 µL 25 mM MgCl_2_, 2.5 µL 10X reaction buffer [(NH_4_)_2_SO_4_], 0.5 µL working stock dNTPs or dNTP mix combined to give a final concentration of 0.2 mM each, 0.25 µL of *Taq* DNA Polymerase (5 U/µL), 15.75 µL of sterilized distilled water and 1 µL of template DNA. Primers 16S-F and 16S-R1 had the sequences of 5′-AAGAGTTTGATCATGGCTCA−3′ and 5′-TAAGGAGGTGATCCAACCGCAGGTTC-3′, respectively. The PCR was performed in a thermal cycler (Bioer XP Cycler) using cycling conditions consisting of an initial denaturation at 95 °C for 3 min, followed by 35 cycles consisting of denaturation at 94 °C for 30 s, annealing at 50 °C for 30 s, and extension at 72 °C for 1 min. A final extension was performed at 72 °C for 5 min. A blank that contained all the components of the reaction mixture without the DNA template was included as a control. The PCR products were analyzed by running on 1% agarose gel electrophoresis.

#### 2.4.3. Agarose Gel Electrophoresis

A 1% (w/v) agarose gel was prepared by mixing 0.25 g of agarose in 25 mL of 1X TAE buffer and placed onto an electrophoresis tank submerged in 1X TAE buffer solution. The loading samples were each prepared by mixing 5 µL of PCR products with 3 µL of 6x loading dye and 1 µL of EZ Vision dye, whereas 1 µL of loading DNA marker or ladder bearing 1.5 kb size was mixed with 2 µL of 6x loading dye, 1 µL of EZ Vision dye, and 5 µL of 1x TAE buffer solution before being run at 90 V and visualized using UV light in a gel imager (Alpha Innotech FluorChem 5500 Gel Imaging System, Cambridge, Massachusetts, USA). A scale-up of PCR products at 50 µL and purification of PCR products were done before sending them out for outsourced sequencing service.

#### 2.4.4. Purification of PCR Products for Sequencing

Direct precipitation is a general method used for the purification of PCR products. A total of 50 µL of PCR products was transferred to a 1.5 mL tube. Sodium acetate (pH 8) at 4 µL and absolute ethanol (99%) at 100 µL were then added, mixed, and kept in ice for 30 min. The tube was centrifuged at 13,040× *g* for 15 min at 4 °C. The supernatant was decanted and 200 µL of 75% ethanol was added and vortex-mixed for 1 min. The tube was centrifuged at 13,040× *g* for 5 min at 4 °C. The pellet was then air-dried and 15 µL of sterile distilled water was added to re-suspend the pellet by tapping. Prior to being sent for sequencing, the quantification or estimation of the concentration of the PCR products within the range of 5 to 100 ng per µL was done using the Gel Imager Software (Alpha Innotech FluorChem 5500 Gel Imaging System, Cambridge, Massachusetts, USA)

#### 2.4.5. Sequencing of 16S rDNA

The determination of the 16S rDNA nucleotide sequences was done by transferring the purified PCR products containing the concentrated amplified DNA, along with primer R2 to First BASE Laboratories Sdn Bhd, a life-sciences-based research company located in Peninsular Malaysia.

#### 2.4.6. Identification of Bacterial Sequences

The sequences obtained for selected bacterial isolates were manually analyzed using Sequence Scanner Software v1.0 by Applied Biosystems^®^ (Forster City, California, USA). The interpretation of the sequences was facilitated by comparing them with information in the BLAST database online (National Centre for Biotechnology Information, Bethesda, Maryland, USA) in which the identification of an isolate is generated with percentages of matched database nucleotide sequences and the calculation of the statistical significance of matches. 

### 2.5. Plant Growth-promoting Assays 

#### 2.5.1. Germination Assays of Maize and Green Gram Seeds

Phytotoxic assays for 15 selected bacterial inoculants on green gram (*Vigna radiata*) seeds obtained from a local market were done via germination check of both root and shoot of the germinating seeds. The seeds were surface-sterilized in 0.1% sodium hypochlorite solution for 5 min and were then rinsed at least three times with sterile distilled water before soaking in broth containing 16 h bacterial cultures, according to treatments, for 2 h at 30 °C. The seed treatments consisted of 15 selected isolate cultures. Two control treatments were included whereby the first control comprised seeds treated with sterile distilled water and the other control comprised treatments with sterile nutrient broth.

The treated green gram seeds were placed on petri dishes containing sterilized moist sheets of Whatman filter paper for each treatment. Each treatment was based on 100 seeds of each plant type. The seeds were incubated for up to 7 days at about 30 °C and moisture content of growth support was maintained with an equal volume of autoclaved distilled water on daily basis. The number of germinated green gram seeds was counted on the third and fourth day of incubation, respectively, followed by a second counting and measurement of the shoots and roots or radicle length for both plants types on the seventh day. The percentage of germinated seeds and the vigor index were calculated. The vigor index was calculated as the sum of the mean shoot and root length multiplied by the percentage of germination [46]. All experimental sets and apparatus used were sterilized where necessary. 

#### 2.5.2. Indole-3-Acetic Acid Production Assay

Some selected bacterial isolates were subjected to an in vitro colorimetric determination of IAA production using Salkowski’s reagent (2% of 0.5 M FeCl_3_ in 35% perchloric acid), as modified from Asghar et al. [47]. Each selected isolate was grown to exponential growth phase (OD ≥ 1.0) within 16 h to be used as pre-inoculum for the bioassay. The inoculum was adjusted to OD_600_ = 0.5 (10^6^ to 10^7^ cfu mL^−1^) before inoculation at 500 µL into 50 mL of nutrient broth without the addition of tryptophan and was incubated at 30 °C under agitation at 100 rpm for up to 48 h. Non-inoculated broth was included as the control. Each culture was centrifuged at 10,000 rpm for 10 min at 4 °C. A total of 1 mL of culture supernatant was mixed with 2 mL of Salkowski’s reagent in a test tube and kept in the dark for 20–25 min before measuring absorbance using a UV-Vis spectrophotometer at 535 nm. The assay for each isolate was conducted in triplicates. Any appearance of a reddish to pinkish color in the solution indicated IAA presence in the cultured medium, and, depending on the color intensity of the test solutions, the coloring responses were classified into two categories: pink type (pale pink to deep pink) and red type (dark red to red). 

### 2.6. Data Analysis of Bioassays

An Analysis of Variance (ANOVA) was used to test for significant differences between measurements of each bioassay, whereas Tukey’s Test was employed to test for significant differences among the different treatments at *p* ≥ 0.05. The Statistical Analysis System (SAS Ver. 9.2, Cary, NC, USA) was used for the analysis.

## 3. Results and Discussion

### 3.1. Functional Activity Assays of Respective Isolates

In this study, 14 out of 62 cellulolytic, 12 out of 39 N-fixing, and 8 out of 36 phosphate-solubilizing bacterial isolates obtained from the rhizospheres of both planted and indigenous tree species in the rehabilitated forest were screened based on their functional activities on their respective selective media and distinct morphology, for further characterization. Among the three functional groups, the phosphate-solubilizing group had the least number of characterized isolates due to the fact that PSB isolates tend to lose their ability after successive transfers or subcultures on agar medium, as has been reported previously [48]. Diazotrophic isolates also encountered a similar phenomenon whereby, upon successive cultivation, some isolates failed to develop blue haloes or were unable to grow on N-free media. A report by da Silva et al. [49] showed that diazotrophic isolates lose their characteristic pellicle growth upon successive cultures, which makes nitrogenase activity assessment not possible. However, none of the cellulolytic isolates tested in this study lost their functional ability.

Cellulolytic microorganisms play a major role in the synthesis of cellulases crucial for the breakdown of complex polysaccharides into simple sugars. There was no specific correlation between cellulose-hydrolyzing efficiency and cellulase (CMCase) activity for the 14 tested cellulolytic strains (Table 1). Among the 14 cellulolytic isolates assessed, isolates C46d and C42d consistently showed higher cellulolytic activity, based on both cellulose-hydrolyzing efficiency (10.41 and 8.52, respectively) and cellulase activity (0.089 U mL^−1^ and 0.077 U mL^−1^, respectively). The cellulase (CMCase) activities of the cellulolytic isolates in this study were comparable to those of bacterial strains isolated from soil under different culture conditions, as reported by Sethi et al. [50]. The cellulase (CMCase) activity demonstrated by C46d was especially high compared to those reported for *Bacillus amyloliquefaciens* SS35 [51] and *Bacillus pumilus* EB3 [52] at 0.079 U mL^−1^. 

For nitrogenase activity assessment, there was a positive relationship (r = 0.63, *p* ≤ 0.05) between the N-fixing efficiency values and BNF amount. Among the 12 diazotrophic isolates evaluated, NB1 exhibited the most active N-fixing activity, as demonstrated by the consistently higher means of both activity efficiency and nitrogenase activity quantification at 10.82 and 2.21 µg N mL^−1^ day^−1^, respectively (Table 2). NB11 also presented comparably high N-fixing activity at 1.87 µg N mL^−1^ day^−1^, although it produced a fairly small halo on the plate assay, yielding a mean efficiency value of 1.88. Strain N20c had the second highest mean for N-fixing efficiency (5.92) but displayed a fairly moderate amount of N-fixing activity (1.24 µg N mL^−1^ day^−1^). Lower N-fixing activity was recorded for the rest of the assessed isolates, with values ranging from 0.34 to 1.38 µg N mL^−1^ day^−1^. Plate assays revealed the lowest N-fixing efficiency values in the range 1.75–2.67. Although there is a lack of reports on N-fixing activity (efficiency) evaluation based on plate assays, the quantitative values were within the range of that for other reported diazotrophic strains [39]. The ability of diazotrophic bacteria to convert atmospheric N_2_ into ammonia, which is the available form of N used by living organisms for biosynthesis, is undeniably of vital importance to soil systems, especially due to the direct influence on plant growth or indirectly due to the production of phytohormones that boost plant growth [49,53]. 

Phosphorus (P) is one of the most limited but important plant growth nutrients. It mainly occurs in insoluble forms, whereby phosphate-solubilizing rhizobacteria play a crucial role in converting P to available forms [54]. Among the eight phosphate-solubilizing strains, PB3 and PB5 showed superior phosphate-solubilization efficiency values in the plate assays (4.21 and 4.10, respectively), whereas, in the enzymatic assays, the highest activity was demonstrated by isolate PC8 (307.7 µg mL^−1^) (Table 3). The amount of phosphate-solubilization efficiency and activities with the final broth culture pH reported in this study is comparable to the range reported elsewhere by Pandey et al. [55], Gulati et al. [56], and Song et al. [57]. Isolate PC8 showed higher phosphate-solubilization activity (307.67 µg mL^−1^) as compared with *Pseudomonas putida* (B0) at the maximum activity of 247 µg mL^−1^ [55]. 

There was no significant relationship between phosphate-solubilizing efficiency and soluble phosphate. Similar observations had been previously reported by Nautiyal [32] and Chang and Yang [58]. Nautiyal [32] reported the inability of phosphate-solubilizing *Pseudomonas aerogenes* and *Pseudomonas* sp.1 to produce haloes on solid media while still being able to solubilize substantial amounts of P in different types of broth cultures. Nonetheless, there was a significant inverse correlation (r = −0.74, *p* ≤ 0.05) between soluble phosphate and the pH of the culture broth, and this observation corroborates previous findings [59,60]. The pH of the broth declined to a low of 4.04, as observed in PC8 activity, from the initial pH 7. This observation is consistent with earlier findings where there was a drop in pH due to bacterial activity accompanied by mineral phosphate-solubilization and increase in activity efficiency [55]. 

### 3.2. Cultural, Biochemical and Molecular Identification of Selected Functional Bacterial Isolates

The isolation of bacteria near the root zones of vegetation thriving in the rehabilitated forest generally leads to the recovery of putative beneficial rhizospheric or even free-living functional bacteria. In this study, growth in the selective media proved to be a successful strategy to select bacteria based on their phenotypic activities on the respective media. Among the isolates assessed for their functional activities, five isolates of each functional group were selected to be further characterized on the basis of these distinct colony morphology and high functional activities. After being selected based on these combined criteria, the isolates were grouped by morphological similarities and phenotypic characteristics.

Based on the morphological characteristics of the 15 selected isolates shown in Appendix A, Table A1, they exhibited varying properties. It was observed that the majority or 13 isolates exhibited round colonies in configuration or form; 10 colonies were convex while the rest showed raised elevations; 14 colonies were opaque in density; colonies of all the isolates were almost creamy or yellowish in color; 13 showed Gram negative reactions; all isolates were rods or short rods in single or in pair arrangements; 13 were without endospore production; and they were mostly motile, as exhibited by 11 isolates. Additionally, the following morphological properties presented more diverse characteristics: colony margin was represented by eight entires, three undulates and serrates, respectively, and one lobate; colony size was represented by six moderates, five smalls, three large, and one pinpoint; and the shapes of the endospores produced by two isolates were rods and coccus, respectively. 

The isolates were primarily identified based on the molecular method, and in cases of multiple identities produced based on the BLAST generated database, morphological (Appendix A, Table A1), and biochemical (Appendix A, Table A2) properties facilitated downsizing of the identification task. The selected cellulolytic isolates in this study were identified as *Serratia nematodiphila* strain SP6 (C46d), *Serratia marcescens* subspecies *sakuensis* isolate PSB23 (C22b), *Stenotrophomonas maltophilia* strain KNUC605 (C29b), *Bacillus thuringiensis* (CB15) and *Stenotrophomonas* sp. Ellin162 (C41d). The N-fixing isolates consisted of *Burkholderia nodosa* strain Br3470 (NB1), *Delftia lacustris* strain 332 (NC9), *Stenotrophomonas* sp. strain SeaH-As3w (N27d), *Ralstonia* sp. strain MCT1 (NB10) and *Cupriavidus basilensis* strain CCUG 49340T (NB11). The phosphate-solubilizing isolates were represented by *Burkholderia cepacia* strain 5.5B (PB5), *Burkholderia cepacia* strain ATCC 35,254 (PB3), *Burkholderia uboniae* (PB7), *Burkholderia cepacia* strain 2EJ5 (PC8) and *Gluconacetobacter diazotrophicus* PAl 5 (PB6). 

These identities were matched with BLAST databases with the range of 97% to 100% base sequence similarities (Table 4). It was observed that identification of bacterial isolates over the three phenotypic groups revealed the predominance of Gram-negative over Gram- positive bacteria in the rehabilitated forest soils. 

The Gram-positive bacteria belonged to only one taxonomic grouping, Firmicutes, represented by the genus *Bacillus*, whereas the Gram-negative bacteria included Alpha-Proteobacteria with the genus *Gluconacetobacter*; Beta-Proteobacteria is represented by the genera *Burkholderia*, *Delftia*, *Ralstonia*, and/or *Cupriavidus*; and Gamma-Proteobacteria is represented by the genera *Serratia* and *Stenotrophomonas*.

From the overall identification results for each functional group of isolates, bacteria from genus *Burkholderia* seemed to predominate in the tropical forest soils with one third of total identified isolates, with distinguishing phosphate-solubilizing and N-fixing capabilities over other genera. The *Stenotrophomonas* genus ranked second in abundance to *Burkholderia*. The *Burkholderia* genus is a well-known classification of bacteria, wherein its members possess vast versatility in niches and nutrition regulation, such as N-fixing and phosphate-solubilization, as well as possessing numerous PGP attributes. *Burkholderia* species occur in the natural environment, even though some strains are also found in clinical samples, especially the *B*. *cepacia* complex (Bcc) species from patients with cystic fibrosis [61].

Based on our 16S rDNA sequence analysis results, the top four isolates with the highest phosphate-solubilizing and N-fixing activities belong to the genus *Burkholderia*, in which three of them were closely related to *B*. *cepacia* (99% similarity) and *B*. *nodosa* (98% similarity), respectively. *Serratia marcescens* (of close similarity by C22b at 97%) or *Serratia* sp. (with C46d closely matched to *Serratia nematodiphila* by 98%) are known to have both cellulolytic and phosphate-solubilizing abilities [62,63]. Similar to the results of this study, *Stenotrophomonas* spp. have been previously reported to possess both N-fixing [64] and cellulose-hydrolyzing abilities [65]. *Stenotrophomonas* species commonly prevail in nature, whereby *S*. *maltophilia* and *S*. *rhizophila* are reported to occur as rhizospheric and endophytic bacteria accountable for beneficial plant interactions [65]. The N-fixing isolates in this study displayed higher diversity in terms of species, as well as activities. 

Diazotrophic isolate N27d revealed a 100% matching identity with *Stenotrophomonas* sp. (strain SeaH-As3w) and it is believed to have cellolulytic potential similar to the C41d and C29b isolates. Previously, many studies have reported bacteria belonging to the genus *Burkholderia,* especially *B*. *cepacia* strains, as having biocontrol ability against many phyto-pathogenic fungi, such as *Fusarium* sp., *Pythium aphanidermatum*, *Pythium ultimum, Phytophthora capsici, Aspergillus flavus, Botrytis cinerea* and *Rhizoctonia solani* [66,67,68,69,70], as part of its main contributing PGP factors. 

The phosphate-solubilizing bacteria showed the lowest diversity with only three species’ identities from two genera recorded among the five identified isolates. Isolates PB3, PB5, PB7, and PC8 were closely matched (99% similarity) to *Burkholderia* sp. These isolates together with the N-fixing NB1 might potentially possess both N-fixing and phosphate-solubilizing activities [71,72]. All *Burkholderia* strains in this study demonstrated variability in cultural and biochemical properties, which is typical for members of this genus. 

The ability of *Burkholderia* strains to solubilize inorganic phosphates has been well documented by various studies [73,74,75,76], a beneficial property in plant growth promotion. Among the *Burkholderia* strains isolated in this study, this trait seemed most prevalent. A few *Burkholderia* strains are known for other PGP abilities, such as the ability to fix N_2_, either as free-living bacteria [77], or even nodulating ones that also fix N_2_ on media, including the *Burkholderia nodosa* strain, as reported by da Silva et al. [78].

The genus *Delftia* (with close similarity of NC9 to *Delftia lacustris* at 99%) belongs to a newly grouped genus closely linked to *Comamonas* [79]. One of the diazotrophic bacterium IHB B 4037, which displayed closely matched identity with *Delftia lacustris* isolated from the rhizosphere of tea plants in India, showed the highest nitrogenase activity in a study conducted by Gulati et al. [80]. *Delftia tsuruhatensis* was also revealed to be a diazotroph with biological control ability against several plant pathogens, especially the three main rice pathogens: the likes of *Xanthomonas oryzae* pv. Oryzae, *Rhizoctonia solani*, and *Pyricularia oryzae* Cavara [81]. In summary, the variability among the bacterial strains of the same genera pointed to the fact that bacterial phenotypic properties are influenced by genetic factors, as well as environmental factors [82].

### 3.3. Seed Germination and Shoot and Root Elongation Assays

Seed germination and shoot and root elongation assays were done on 15 selected functional isolates on Green Gram seeds (Table 5) to represent an attribute of early plant growth promotion activity by the isolates. 

After a week of growth, Green Gram roots originating from seeds treated with all selected bacterial strains exhibited different effects on root lengths, shoot lengths, and seedling vigor indexes compared with the two controls, treated with distilled water and nutrient broth, respectively. As shown in Table 5, inoculation with cellulolytic isolate C41d (*Stenotrophomonas* sp.) resulted in significantly longer root lengths compared to both control treatments but was not significantly different from the rest of the cellulolytic isolates. On the other hand, the treatments with all selected cellulolytic strains resulted in significantly higher shoot lengths compared to both controls but were not different from one another, ranging from 11.46 to 12.9 cm. In terms of the seedling vigor index (SVI), all cellulolytic strains exhibited significantly higher values, at least 33.6% to 42.7% higher than both controls, with isolate C22b (*Serratia marcescens* subsp. *sakuensis*) showing outstanding values but not different from isolates CB15, C41d, and C46d (Bt, *Stenotrophomonas* sp., and *Serratia nematodiphila*), which ranged from 1670 to 1815. 

The treatments with N-fixing isolates NC9 (*Delftia lacustris*) and NB11 (*Cupriavidus basilensis*) showed higher root lengths and shoot lengths occurring in the range of 3.76 cm to 4.44 cm and 10.68 cm to 11.33 cm, respectively, whereas the SVI values for isolates NB1, NC9, and NB10 (*Burkholderia nodosa*, *Delftia lacustris* and *Ralstonia* sp.) revealed significantly higher SVI values than both controls, ranging from 1444 to 1576, that is, at least 16.5% to 19.8% higher but not significantly different from the rest of the N-fixing strains (Table 5). 

All the five selected phosphate-solubilizing isolates resulted in significantly higher SVI values than both controls by at least 14.4% to 37.9%, that is, in the range of 1429 to 1748. Isolates PB3, PB5, and PB7 (*Burkholderia cepacia*, *Burkholderia cepacia*, and *Burkholderia uboniae*) resulted in the longest root lengths among the phosphate-solubilizing strains in the range of 4.26 to 5.16 cm. However, significantly higher shoot lengths were achieved with phosphate-solubilizing isolates than both controls, ranging from 10.53 to 12.33 cm. Isolate PB3 had the most outstanding effect on shoot length, but was not different from that of isolates PB5 and PB7, among the other PSB strains. (Table 5).

A previous study by Satya et al. [83] resulted in similar observations for *Burkholderia* sp. as their application using *Burkholderia* sp. strain TNAU-1 demonstrated improvement in root and shoot lengths, as well as increased seedling vigour in mung beans. According to Egamberdieva et al. [84], the inoculation of one of the salt tolerant rhizobacterial strains, *Stenotrophomonas rhizophila* ep-17, resulted in the improved dry weight of cucumber by up to 68% in various salt concentrations as high as 10 dSm^−1^ in comparison to non-inoculated plants exposed to salt stress. 

Hameeda et al. [85] demonstrated that one of the phosphate-solubilizing bacterial strains in their study, *Serratia marcescens*, designated as EB 67, which was tested on maize growth by the paper towel method, was able to produce higher root and plumule lengths, plant weight (43%), and seed vigor index, whereas another strain also included in the study, *Serratia* sp., designated as EB75, resulted in increased root length, plant weight by 23%, and seed vigor index over that of the uninoculated control.

Kang et al. [86] reported that the inoculation of a novel cold stress resistant rhizobacteria *Serratia nematodiphila* PEJ1011 on pepper plants resulted in the latter’s significantly improved growth in terms of both increased root and shoot lengths and fresh weight of the peppers in both normal and cold temperatures as compared with controls under normal and cold temperature, respectively. Apart from *Bacillus thuringiensis* (Bt), which has been reported as having apparent biological control attributes, a study by Khan et al. [87] also reported plant growth-promotion via an increase in the plant growth parameters of mung bean seeds coated with various Bt strains used in their study. Apart from having high nitrogenase activity strains from rhizospheric soil of tea plants [80,88], *Delftia lacustris* isolated from rhizospheric soils of tobacco was found to be a strongly antagonistic bacteria against the pathogen *Phytophthora nicotianae*, which could have potentially helped in promoting growth of the tobacco crop. 

*Cupriavidus* (synonym *Ralstonia*) is closely linked to *Burkholderia*, but, compared to the latter genus, it consists of very limited N-fixing species [89]. Both genera, *Burkholderia* spp. and *Cupriavidus taiwanensis*, have been reported to be N-fixing symbionts of legume *Mimosa* with the ability to nodulate the latter [90,91,92]. 

*Burkholderia nodosa* designated as LMG 23,741 has also been isolated from acidic soils of the Amazon as a nodulating N-fixing bacteria [78], as compared to the strain in this study that did not nodulate but was a free-living or rhizospheric N fixer. Both strains are known to be unable to solubilize inorganic phosphates. The strain reported by da Silva et al. [78] also did not possess anti-fungal characteristics. Besides the N-fixing isolate *Ralstonia* sp. characterized in this study, *Ralstonia taiwanensis* is the first N-fixing beta-proteobacterium strain with the ability to nodulate the roots of *Mimosa* from which it was isolated [93]. 

*Burkholderia cepacia* designated as LMG 1222 by da Silva et al. [78] was also reported as a non-N-fixing bacteria isolated from acidic Amazon soils that could solubilize inorganic phosphates, in addition to possessing anti-fungal properties. However, the literature is lacking on the beneficial properties of *Burkholderia uboniae*, and, hence, the results of this study could contribute to the primary evaluation on the potential of this particular strain on early plant growth promotion. *Cupriavidus basilensis* designated HMF14 has also been recently identified as an important furan-degrading bacterium and was isolated by employing an elegant assay based on the germination of lettuce seeds [94].

*Gluconacetobacter diazotrophicus* is one of the diazotrophic species reported to form anomalous symbiotic relationships with plants by thriving on the root system surface as rhizobacteria, whereas a number of strains are known to infect plant tissues as endophytic bacteria and to fix N_2_, resulting in enhanced plant growth [95,96,97,98].

### 3.4. Indole-3-Acetic Acid Assay

Out of the 15 previously selected functional bacterial isolates for the IAA assay, only seven isolates produced IAA, as shown in Table 6.

All the seven selected strains produced IAA without supplementation of L-tryptophan, from N-fixing isolate *Burkholderia nodosa* NB1 to the most abundant IAA production by cellulolytic isolates *Stenotrophomonas maltophilia* C29b and *Serratia nematodiphila* C46d, and phosphate-solubilizing isolate *Burkholderia cepacia* PC8, ranging from 8.17 to 13.86 µg mL^−1^. The IAA produced by the cellulolytic, non-N-fixing *Stenotrophomonas maltophilia* strain in this study, C29b, was 12.38 µg mL^−1^, in the absence of tryptophan. *Stenotrophomonas maltophilia* has been previously reported as being diazotrophic with IAA production of 2.6 mg mL^−1^ [99], 112.8 µg mL^−1^ for S. *maltophilia* PM-1, and 139.2 µg mL^−1^ in *S*. *maltophilia* PM-26 [100] with L-tryptophan as a supplement. 

The *Serratia nematodiphila* C46d strain in this study was found to synthesize IAA, as well as solubilize phosphate at 11.39 µg mL^−1^ and 14.25 µg mL^−1^, respectively. In comparison, *Serratia nematodiphila* NII-0928 isolated from Western ghat forest soil was also found to possess multiple PGP attributes, including phosphate-solubilization, and IAA production at higher concentrations, with values of 76.6 µg mL^−1^ and 58.9 µg mL^−1^, respectively [101]. As stated previously, the variability of the bacterial strains of the same genera points to the fact that bacterial phenotypic properties are influenced by genetic and environmental factors [82]. The *Bacillus thuringiensis* strain CB15 in this study yielded IAA quantities within the range of 0.12 to 6.59 µg mL^−1^, as was reported by Vidal-Quist et al. [102]. 

*Burkholderia cepacia* strains PC8 and PB3 evaluated in this study, without the addition of tryptophan, produced IAA quantities of 8.45 and 7.81 µg mL^−1^, respectively. Bevivino et al. [103] reported that rhizospheric strains of *Burkholderia cepacia* PHP7 and TVV75 were found to produce IAA of approximately 0.6 and 0.4 µg mL^−1^, respectively, both of which were far lacking by at least 14-folds, even with the addition of L-tryptophan, in comparison to the strains in this study. This phenomenon could indicate that *B*. *cepacia* strains are actively involved in IAA synthesis in pure cultures [104]. 

The phosphate-solubilizing and non-N-fixing *Gluconacetobacter diazotrophicus* strain PB6 produced an IAA quantity of 7.51 µg mL^−1^ without the precursor tryptophan, which was higher than that reported previously, and improved the shoot length and vigor index of green gram compared to that of the uninoculated controls. Anitha and Thangaraju [105] reported an endophytic diazotroph *Gluconacetobacter diazotrophicus* strain in their study to have exhibited nitrogenase activity, phosphate-solubilization, and IAA production with and without tryptophan, with values of 4.74 µg mL^−1^ and 1.4 µg mL^−1^, respectively. Despite the lesser amount of IAA produced by the *Burkholderia nodosa* strain NB1 with a value of 0.43 µg mL^−1^, this is the first result on IAA production ever reported for the non-nodulating N-fixing *Burkholderia nodosa* strain. 

The presence of an appropriate precursor such as L-tryptophan may mediate the production of auxins by some microorganisms [106]. The influence of auxins on plant seedlings depends on their concentrations. For instance, high concentrations may suppress growth, whereas low concentrations may produce stimulative effects on growth [107]. The absence of tryptophan precursor in IAA production has also been previously observed in other bacterium, such as *Azotobacter brasilense,* in a mechanism known as a tryptophan-independent pathway, which was suggested during experiments using labelled tryptophan [108]. 

According to Spaepen and Vanderleyden [109], the occurrence of some IAA production, despite the inactivation of every pathway, in some studies revealed the redundancy of IAA biosynthetic pathways in some of the studied microorganisms, indicating that several pathways do exist and are active in a single microorganism. Nevertheless, the ability to secrete IAA without absolute dependence on tryptophan as the main precursor proved to be an added advantage over tryptophan dependent microbes in plant rhizospheres to produce beneficial associative effects on plant roots to enhance plant growth.

## 4. Conclusions

The results of this study reveal the presence of some efficient and beneficial cellulolytic, N-fixing, phosphate-solubilizing bacterial species among the natural rhizo-bacterial community in soils of a tropical rehabilitated forest at Universiti Putra Malaysia Bintulu Campus, Malaysia. Out of the 15 identified bacterial isolates possessing beneficial phenotypic traits, a third belong to the genus *Burkholderia* and a fifth belong to *Stenotrophomonas* sp., with both genera consisting of members from two different functional groups. The results of the experiments also confirm the multiple PGP traits of the selected bacterial isolates based on their respective functional activities, root and shoot lengths, and seedling vigour improvements when bacterized on mung bean seeds, as well as significant IAA production. Some of the results were comparable, if not better in some cases, to those of related studies. All these functional bacterial strains could potentially produce beneficial synergistic effects via their versatile properties to improve soil fertility and possible plant growth stimulation through beneficial interactions. 

## Figures and Tables

**Table 1:** Cellulose-hydrolyzing efficiency and cellulase (CMCase) activities (U mL^−1^ cell-free culture broth after 5 days at 30 °C, 180 rpm, and medium pH 7) of cellulolytic isolates.

Isolates	Activity Efficiency	Cellulase Activity (U mL^−1^)
Mean Value ± S.E.		
C14	5.25 ^b,c^ ± 1.50	0.016 ^f^ ± 0.002
C39d	5.10 ^b,c^ ± 0.44	0.015 ^f^ ± 0.002
C41d	5.29 ^b,c^ ± 0.88	0.078 ^b^ ± 0.001
C42d	8.52 ^a,b^ ± 1.93	0.077 ^b^ ± 0.001
C46d	10.41 ^a^ ± 0.43	0.089 ^a^ ± 0.001
C22b	10.31 ^a^ ± 0.62	0.035 ^e^ ± 0.001
C34c	4.57 ^c^ ± 3.24	0.047 ^d^ ± 0.001
C45d	5.93 ^b,c^ ± 1.23	0.059 ^c^ ± 0.001
C4	3.59 ^c^ ± 0.14	0.079 ^b^ ± 0.002
C25b	3.70 ^c^ ± 0.79	0.078 ^b^ ± 0.001
C29b	5.57 ^b,c^ ± 0.70	0.080 ^b^ ± 0.001
C40d	4.63 ^c^ ± 0.61	0.077 ^b^ ± 0.001
C43d	4.60 ^c^± 2.26	0.078 ^b^ ± 0.001
CB15	5.54 ^b,c^ ± 0.36	0.060 ^c^ ± 0.001

S.E. is standard error of the mean. Means with the same letter are not significantly different at *p* ≥ 0.05 within columns using Tukey’s test.

**Table 2:** Nitrogen-fixation efficiency and the BNF quantification of diazotrophic isolates.

Isolate	Activity Efficiency	Quantitative Activity (µg N mL^−1^ day^−1^)
Mean Value ± S.E.		
NB1	10.82 ^a^ ± 0.26	2.21 ^a^ ± 0.34
NB4	1.84 ^e^ ± 0.05	1.11 ^c,d^ ± 0.27
NB5	2.03 ^e^ ± 0.06	0.65 ^c,d^ ± 0.00
NB7	1.97 ^e^ ± 0.05	1.23 ^b,c^ ± 0.24
NB8	1.80 ^e^ ± 0.05	0.47 ^d^ ± 0.00
NC9	4.63 ^c^ ± 0.12	0.84 ^c,d^ ± 0.00
NB10	2.35 ^e^ ± 0.32	1.01 ^c,d^ ± 0.19
NC11	1.95 ^e^ ± 0.04	0.44 ^d^ ± 0.10
NB11	1.88 ^e^ ± 0.07	1.87 ^a,b^ ± 0.34
N20c	5.92 ^b^ ± 0.46	1.24 ^b,c^ ± 0.30
N27d	3.61^d^ ± 0.21	0.73 ^c,d^ ± 0.00
N29d	1.94 ^e^ ± 0.08	0.60 ^c,d^ ± 0.00

S.E. is standard error of the mean. Means with the same letter are not significantly different at *p* ≥ 0.05 within columns using Tukey’s test.

**Table 3:** Phosphate-solubilization efficiency and quantitative phosphate-solubilization estimation based on soluble P by phosphate-solubilizing isolates.

Isolate	Phosphate-solubilizing Efficiency	Quantitative Phosphate-Solubilization (Soluble P in µg mL^−1^)	Final Mean pH in Broth Culture
Mean Value ± S.E.			
PB5	4.21 ^a^ ± 0.22	96.00 ^d^ ± 2.65	4.27
PC1	2.38 ^c,d^ ± 0.15	9.14 ^f^ ± 0.75	4.93
PB7	3.18 ^b^ ± 0.23	159.00 ^c^ ± 1.53	4.15
PB3	4.10 ^a^ ± 0.23	209.17 ^b^ ± 8.97	4.11
PC6	1.85 ^e,f^ ± 0.05	31.53 ^e^ ± 0.39	4.63
PC8	2.74 ^b,c^ ± 0.22	307.67 ^a^ ± 8.41	4.04
PB11	1.66 ^f^ ± 0.08	3.25 ^f^ ± 0.52	4.57
PB6	2.19 ^d,e^ ± 0.24	12.67 ^f^ ± 0.55	4.20

S.E. is standard error of the mean. Means with the same letter are not significantly different at *p* ≥ 0.05 within columns using Tukey’s test.

**Table 4:** Identities of cellulolytic, nitrogen-fixing, phosphate-solubilizing bacterial isolates based on 16S rDNA analysis and the BLAST databases.

Phenotype	Isolate	Closest Identity	Similarity (%)	Accession No.
**Cellulolytic**	C46d	*Serratia nematodiphila* strain SP6	98 (324/329)	JN315887.1
	C22b	*Serratia marcescens* subspecies *sakuensis* isolate PSB23	97 (578/594)	HQ242736.1
	C29b	*Stenotrophomonas maltophilia* strain KNUC605	99 (945/949)	HM047520.1
	CB15	*Bacillus thuringiensis*	99 (1018/1021)	FR846529.1
	C41d	*Stenotrophomonas* sp. Ellin162	100 (989/989)	AF409004.1
**N-fixing**	NB1	*Burkholderia nodosa* strain Br3470	98 (943/967)	AM284972.1
	NC9	*Delftia lacustris* strain 332	99 (1009/1014)	EU888308.1
	N27d	*Stenotrophomonas* sp. SeaH-As3w	100 (1029/1029)	FJ607350.1
	NB10	*Ralstonia* sp. MCT1	99 (999/1007)	DQ232889.1
	NB11	*Cupriavidus basilensis* strain CCUG 49340T	99 (961/969)	FN597608.1
**Phosphate-solubilizing**	PB5	*Burkholderia cepacia* strain 5.5B	99 (874/885)	FJ652617.1
	PB3	*Burkholderia cepacia* strain ATCC 35254	99 (990/993)	AY741346.1
	PB7	*Burkholderia uboniae*	99 (993/997)	AB030584.1
	PC8	*Burkholderia cepacia* strain 2EJ5	99 (1008/1016)	GQ383907.1
	PB6	*Gluconacetobacter diazotrophicus* PAl 5	97 (778/801)	CP001189.1

**Table 5:** Effects of selected cellulolytic, N-Fixing, phosphate-solubilizing bacterial inoculation on the early growth stage of green gram.

Treatment	Mean Number of Seed Germinated (3rd day)	Mean Number of Seed Germinated (7th day)	Mean Length of Shoots (cm) (Mean ± S.E.)	Mean Length of Radicle (cm) (Mean ± S.E.)	% Germinated Seeds	Vigor index (Mean ± S.E.)
**Cellulolytic**						
**C22b**	50.0	50.0	12.84 ^a^ ± 0.06	5.16 ^a,b^ ± 0.04	100	1799.50 ^a^ ± 2.50
**CB15**	50.0	50.0	12.38 ^a^ ± 0.45	5.47 ^a,b^ ± 0.75	100	1785.00 ^a,b^ ± 30.00
**C41d**	50.0	50.0	11.75 ^a^ ± 0.03	5.67 ^a^ ± 0.13	100	1741.50 ^a,b^ ± 10.50
**C46d**	50.0	50.0	11.73 ^a^ ± 0.23	5.28 ^a,b^ ± 0.53	100	1700.00 ^a,b^ ± 30.00
**C29b**	50.0	50.0	11.81 ^a^ ± 0.35	5.04 ^a,b^ ± 0.40	100	1684.50 ^b^ ± 5.50
**Control (Nutrient broth)**	50.0	50.0	9.26 ^b^ ± 0.18	3.36 ^b,c^ ± 0.02	100	1261.00 ^c^ ± 19.00
**Control (dH_2_O)**	49.5	49.5	7.91 ^b^ ± 0.18	2.87 ^c^ ± 0.10	99	1067.00 ^d^ ± 17.00
**N-fixing**						
**NB1**	50.0	50.0	11.27 ^a^ ± 0.06	3.84 ^a,b^ ± 0.02	100	1510.00 ^a^ ± 4.00
**NC9**	50.0	50.0	11.00 ^a^ ± 0.32	4.10 ^a^ ± 0.34	100	1510.00 ^a^ ± 66.00
**NB10**	50.0	50.0	10.81 ^a,b^ ± 0.15	3.89 ^a,b^ ± 0.24	100	1469.00 ^a^ ± 9.00
**NB11**	50.0	50.0	10.43 ^a,b^ ± 0.27	4.09 ^a^ ± 0.26	100	1451.50 ^a,b^ ± 52.50
**N27d**	50.0	50.0	10.31 ^a,b^ ± 0.61	3.68 ^a,b^ ± 0.19	100	1399.00 ^a,b^ ± 42.00
**Control (Nutrient broth)**	50.0	50.0	9.26 ^bc^ ± 0.18	3.36 ^a,b^ ± 0.02	100	1261.00 ^b,c^ ± 19.00
**Control (dH_2_O)**	49.5	49.5	7.91^c^ ± 0.18	2.87 ^b^ ± 0.10	99	1067.00 ^c^ ± 17.00
**Phosphate-solubilizing**						
**PB3**	50.0	50.0	12.25 ^a^ ± 0.08	5.14 ^a^ ± 0.02	100	1738.50 ^a^ ± 9.50
**PB7**	50.0	50.0	12.08 ^a,b^ ± 0.40	4.89 ^a,b^ ± 0.33	100	1696.00 ^a,b^ ± 7.00
**PB5**	50.0	50.0	11.78 ^a,b,c^ ± 0.21	4.61^a,b^ ± 0.35	100	1638.50 ^b^ ± 14.50
**PB6**	50.0	50.0	10.97 ^b,c^ ± 0.06	4.18 ^a,b,c^ ± 0.31	100	1514.50 ^c^ ± 25.50
**PC8**	50.0	50.0	10.71 ^c^ ± 0.18	3.73 ^b,c,d^ ± 0.04	100	1443.00 ^c^ ± 14.00
**Control (Nutrient broth)**	50.0	50.0	9.26 ^d^ ± 0.18	3.36 ^c,d^ ± 0.02	100	1261.00 ^d^ ± 19.00
**Control (dH_2_O)**	49.5	49.5	7.91 ^e^ ± 0.18	2.87 ^d^ ± 0.10	99	1067.00 ^e^ ± 17.00

Data are presented as mean ± standard error (S.E.) of the duplicates of 50 seedlings for each replicate per treatment for root elongation assay and seedling vigor index. The roots emerging from seeds failing to germinate after two days were previously marked and were not measured. The seedling vigor index (SVI) was calculated using the formula: SVI = % of germination x seedling length (root length + shoot length). In the same column, significant differences according to Tukey’s test at the *p* ≤ 0.05 level are indicated by different letters.

**Table 6:** Indole-3-acetic acid production by selected functional bacterial isolates.

Phenotype	Isolate	IAA Production (µg mL^−1^) Mean ± S.E.
	*Stenotrophomonas maltophilia* C29b	12.38 ^a^ ± 1.48
**Cellulolytic**	*Serratia nematodiphila* C46d	11.39 ^a,b^ ± 1.68
	*Bacillus thuringiensis* CB15	0.98 ^c^ ± 0.16
	*Burkholderia cepacia* PC8	8.45 ^a,b^ ± 0.28
**Phosphate-solubilizing**	*Burkholderia cepacia* PB3	7.81 ^b^ ± 0.11
	*Gluconacetobacter diazotrophicus* PB6	7.51 ^b^ ± 0.17
**Nitrogen-fixing**	*Burkholderia nodosa* NB1	0.43 ^c^ ± 0.45

S.E. is the standard error of the mean value. Significant differences according to Tukey’s test at *p* ≤ 0.05 level are indicated by different letters.

## Appendix A

**Table A1:** Morphological characteristics of selected functional isolates.

Colony Morphology	Isolate														
	CB15	C22d	C29b	C41d	C46d	NB1	NB10	NB11	N27d	NC9	PB3	PB5	PB6	PB7	PC8
**Configuration/Form**	Irregular	Round	Round	Irregular	Round	Round	Round	Round	Round	Round	Round	Round	Round	Round	Round
**Margin**	Undulate	Entire	Entire	Serrate	Entire	Entire	Undulate	Undulate	Entire	Entire	Entire	Serrate	Serrate	Entire	Lobate
**Elevation**	Raised	Convex	Convex	Raised	Convex	Convex	Raised	Convex	Convex	Convex	Convex	Raised	Raised	Convex	Convex
**Density**	Opaque	Opaque	Opaque	Opaque	Opaque	Opaque	Opaque	Opaque	Opaque	Opaque	Opaque	Opaque	Transparent	Opaque	Opaque
**Pigments**	Cream	Cream	Yellow	Cream	Cream	Cream	Cream	Cream	Yellowish cream	Cream	Yellowish cream	Cream	Cream	Cream	Cream
**Gram’s reaction**	+	−	−	−	−	−	−	−	−	−	−	−	+	−	−
**Shape**	Rods	Rods	Rods	Short rods	Short rods	Short rods	Short rods	Rods	Short rods	Short rods	Rods	Rods	Short rods	Rods	Short rods
**Size**	Large	Moderate	Small	Large	Small	Pinpoint	Large	Moderate	Small	Moderate	Moderate	Moderate	Moderate	Small	Small
**Arrangement**	Single	Single	Single/pairs	Single	Single	Single	Single	Single	Single	Single/pairs	Single	Single/pairs	Single/pairs	Single/pairs	Single/pairs
**Endospore production**	+	−	−	−	−	−	−	−	+	−	−	−	−	−	−
**Endospore shape**	Rods	−	−	−	−	−	−	−	Coccus	−	−	−	−	−	−
**Motility**	+	+	+	−	+	−	+	−	−	+	+	+	+	+	+

Note: +, positive result; −, negative result.

**Table A2:** Biochemical properties of selected functional bacterial isolates.

Isolate	Catalase	MCA	KIA	SA	SCA	SMA	T80A	NG	Indole	NRT
**CB15**	+	˗	Glu ferm +, Lacferm −	˗	˗	+	+	+	˗	+
**C22d**	+	+	Glu ferm +, Lacferm −	˗	+	+	+	+	˗	+
**C29b**	+	+	Glu ferm −, Lacferm −	˗	+	+	+	+	˗	+
**C41d**	+	˗	Glu ferm +, Lacferm −	˗	˗	+	˗	+	˗	+
**C46d**	+	+	Glu ferm +, Lacferm −	˗	+	+	+	+	˗	+
**NB1**	+	+	Glu ferm −, Lacferm −	˗	+	˗	+	˗	˗	+
**NB10**	+	˗	Glu ferm +, Lacferm −	+	+	+	+	+	˗	˗
**NB11**	+	˗	Glu ferm −, Lacferm +	+	˗	˗	+	˗	˗	+
**N27d**	+	+	Glu ferm −, Lacferm −	˗	+	+	˗	+	˗	+
**NC9**	+	+	Glu ferm +, Lacferm −	˗	+	+	+	+	˗	+
**PB3**	+	+	Glu ferm −, Lacferm −	˗	+	+	+	+	˗	+
**PB5**	+	˗	Glu ferm −, Lacferm −	+	+	+	˗	+	˗	+
**PB6**	+	˗	Glu ferm −, Lacferm −	+	+	+	˗	+	˗	+
**PB7**	+	+	Glu ferm −, Lacferm −	˗	+	+	+	+	˗	+
**PC8**	+	+	Glu ferm −, Lacferm −	˗	+	+	+	+	˗	+

Note: +, positive result; −, negative result; MCA: Mac Conkey agar assay; KIA: Kligler Iron Agar assay; Glu ferm, Glucose fermentation; Lacferm, Lactose fermentation; SA: Starch agar assay; SCA: Simmons citrate agar; SMA: Skim milk agar; T80A: Tween 80 Agar assay; NG: Nutrient gelatine assay; Indole: Indole production assay; NRT: Nitrate reduction test assay.
